# Associação entre Comportamentos de Autocuidado e o Controle Glicêmico Inadequado em Participantes do ELSA-Brasil com Diabetes Tipo 2

**DOI:** 10.36660/abc.20240867

**Published:** 2025-06-27

**Authors:** Gisele de Souza Tupinambá, Odaleia Barbosa de Aguiar, Bruno Pereira de Moura, Maria Inês Schmidt, Maria Del Carmen Bisi Molina, Rosane Harter Griep, Maria de Jesus Mendes da Fonseca

**Affiliations:** 1 Fundação Oswaldo Cruz Escola Nacional de Saúde Pública Sérgio Arouca Programa de Epidemiologia em Saúde Pública Rio de Janeiro RJ Brasil Fundação Oswaldo Cruz – Escola Nacional de Saúde Pública Sérgio Arouca – Programa de Epidemiologia em Saúde Pública, Rio de Janeiro, RJ – Brasil; 2 Universidade do Estado do Rio de Janeiro Instituto de Nutrição Departamento de Nutrição Aplicada Rio de Janeiro RJ Brasil Universidade do Estado do Rio de Janeiro – Instituto de Nutrição – Departamento de Nutrição Aplicada, Rio de Janeiro, RJ – Brasil; 3 Universidade Federal do Rio Grande do Sul Programa de Pós-Graduação em Epidemiologia Porto Alegre RS Brasil Universidade Federal do Rio Grande do Sul – Programa de Pós-Graduação em Epidemiologia, Porto Alegre, RS – Brasil; 4 Universidade Federal do Espírito Santo Programa de Pós-Graduação em Nutrição e Saúde Vitória ES Brasil Universidade Federal do Espírito Santo – Programa de Pós-Graduação em Nutrição e Saúde, Vitória, ES – Brasil; 5 Fundação Oswaldo Cruz Instituto Oswaldo Cruz Laboratório de Educação em Ambiente e Saúde Rio de Janeiro RJ Brasil Fundação Oswaldo Cruz – Instituto Oswaldo Cruz – Laboratório de Educação em Ambiente e Saúde, Rio de Janeiro, RJ – Brasil; 6 Fundação Oswaldo Cruz Escola Nacional de Saúde Pública Sérgio Arouca Departamento de Epidemiologia e Métodos Quantitativos em Saúde Rio de Janeiro RJ Brasil Fundação Oswaldo Cruz – Escola Nacional de Saúde Pública Sérgio Arouca – Departamento de Epidemiologia e Métodos Quantitativos em Saúde, Rio de Janeiro, RJ – Brasil

**Keywords:** Diabetes Mellitus Tipo 2, Autocuidado, Controle Glicêmico, Adesão à Medicação, Alimento Processado

## Abstract

**Fundamento:**

O diabetes tipo 2 (DT2) é uma condição crônica prevalente, frequentemente associada a complicações metabólicas e cardiovasculares.

**Objetivo:**

Este estudo objetivou avaliar a associação entre comportamentos de autocuidado, como consumo de ultraprocessados, ingestão de legumes, verduras e frutas, prática de atividade física, adesão ao tratamento farmacológico e tabagismo, e o controle glicêmico inadequado em participantes do ELSA-Brasil vivendo com DT2.

**Métodos:**

Este é um estudo transversal com 2567 indivíduos, no qual as associações entre as variáveis de interesse foram analisadas por regressão logística múltipla (p<0,05).

**Resultados:**

A adesão ao tratamento farmacológico foi o único comportamento consistentemente associado ao controle glicêmico inadequado. Participantes com baixa adesão apresentaram mais que o dobro de chances de terem níveis inadequados de hemoglobina glicada (OR: 2,09; IC 95%: 1,14–4,10). O consumo de ultraprocessados e a ingestão insuficiente de vegetais mostraram associações iniciais que não se mantiveram significativas após ajustes, sugerindo a influência de fatores adicionais, como condições socioeconômicas.

**Conclusão:**

Esses resultados reforçam a necessidade de intervenções integradas que combinem educação em saúde, suporte ao autocuidado e políticas públicas voltadas à equidade no acesso a tratamentos, contribuindo para o controle glicêmico e a redução das complicações associadas ao DT2.

## Graphical abstract

**Figura Central:**
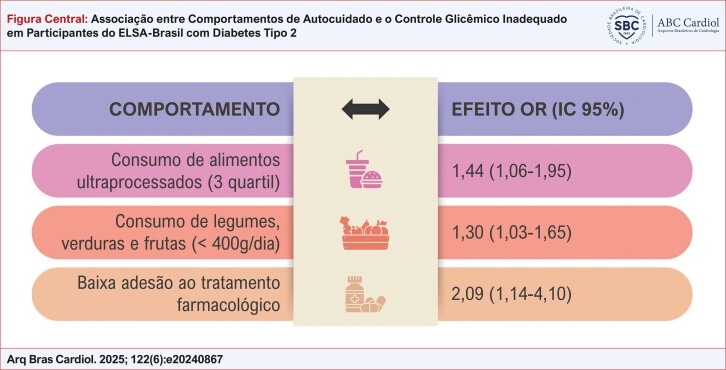
: Associação entre Comportamentos de Autocuidado e o Controle Glicêmico Inadequado em Participantes do ELSA-Brasil com Diabetes Tipo 2 Tamanho do efeito de cada modelo final para os diferentes comportamentos de autocuidado em relação ao controle glicêmico inadequado em participantes do ELSA-Brasil diagnosticados com diabetes tipo 2, 2012-2014.

## Introdução

O diabetes tipo 2 (DT2) é uma condição crônica de alta prevalência mundial, associada a diversas complicações metabólicas e cardiovasculares que impactam a qualidade de vida e a mortalidade da população.^1,2^ Estima-se que, globalmente, mais de 529 milhões de pessoas vivam com diabetes, sendo que o DT2 corresponde a aproximadamente 96% dos casos de diabetes.^3^ No Brasil, o cenário é igualmente preocupante, com uma prevalência crescente nos últimos anos, sobretudo em adultos de meia-idade e idosos, demandando estratégias eficazes para o controle e manejo da doença.^4^

O autocuidado é uma estratégia importante para o tratamento do diabetes e pode contribuir para o controle glicêmico adequado, para a redução das complicações da doença e a melhora na qualidade de vida.^5^ A hemoglobina glicada (A1C) é um marcador amplamente utilizado para avaliar o controle glicêmico em indivíduos com diabetes, pois reflete os níveis médios de glicose sanguínea nos últimos dois a três meses.^6^ Segundo as diretrizes mais atuais, uma A1C inferior a 7% é considerada adequada, enquanto valores iguais ou superiores a 7% indicam controle glicêmico inadequado, o que aumenta o risco de complicações micro e macro vasculares.^7,8^

O manejo adequado do DT2 envolve não apenas a administração de medicamentos hipoglicemiantes, mas também a adoção de um conjunto de comportamentos de autocuidado que são essenciais para o controle glicêmico e a prevenção de complicações.^9^ De acordo com a literatura, os principais comportamentos de autocuidado incluem a prática regular de atividade física, a adesão ao tratamento farmacológico, o consumo alimentar balanceado, e a abstinência de tabagismo.^10,11^ Além disso, o consumo de alimentos ultraprocessados, amplamente difundidos na sociedade contemporânea, tem sido associado ao pior controle metabólico,^12^ enquanto a ingestão de alimentos ricos em fibras, como legumes, verduras e frutas, contribui positivamente para o controle da glicemia.^13,14^

Estudos que avaliem esses comportamentos de autocuidado em grandes populações são escassos no Brasil.^13,15,16^ O Estudo Longitudinal da Saúde do Adulto (ELSA-Brasil)^17^ é uma coorte multicêntrica que oferece uma oportunidade única para investigar essa relação em uma amostra de grande escala, permitindo explorar como diferentes práticas de autocuidado influenciam o controle glicêmico em indivíduos diagnosticados com DT2.

Dado o impacto significativo dos comportamentos de autocuidado no manejo do DT2 e a necessidade de melhor compreensão das variáveis que influenciam o controle glicêmico, este estudo tem como objetivo avaliar a associação entre comportamentos de autocuidado (consumo de ultraprocessados, consumo de legumes, verduras e frutas, prática de atividade física, adesão ao tratamento farmacológico e tabagismo) e o controle glicêmico inadequado em participantes do ELSA-Brasil com DT2.

## Métodos

### Desenho do estudo e amostra

Este é um estudo observacional seccional que utilizou dados da segunda onda (2012-2014) do ELSA-Brasil, um estudo coorte multicêntrico em andamento, que investiga a incidência e os fatores de risco para doenças crônicas em uma população brasileira.^17^ Em sua linha de base (2008-2010), foram coletados dados de 15105 funcionários ativos e aposentados, de ambos os sexos, com idades entre 35 e 74 anos, provenientes de seis instituições públicas localizadas em diferentes estados do Brasil – Fundação Oswaldo Cruz (FIOCRUZ - Rio de Janeiro), Universidade de São Paulo (USP), e as Universidades Federais da Bahia (UFBA), Espírito Santo (UFES), Minas Gerais (UFMG) e Rio Grande do Sul (UFRGS).

Como critério de inclusão para o presente estudo, os participantes deveriam possuir diagnóstico de DT2, o qual foi determinado com base nas seguintes informações – diabetes autorreferido, uso autorreferido de medicamentos para diabetes nas duas semanas anteriores à coleta dos dados ou exames laboratoriais alterados (glicemia de jejum ≥ 126 mg/dL, A1C ≥ 6,5% ou glicemia 2 horas após sobrecarga ≥ 200 mg/dL pós carga).^18^ O critério de exclusão incluiu os participantes que haviam se submetido a cirurgia bariátrica. Estudos mostram que pacientes pós-cirúrgicos tendem a aumentar o consumo de alimentos ultraprocessados nos fins de semana em comparação aos dias úteis, enquanto diminuem a ingestão de frutas e vegetais.^19,20^ Dos 14 014 indivíduos da segunda onda do ELSA-Brasil, foram selecionados 2567 participantes com diagnóstico de DT2 para compor a amostra deste estudo.

Por se tratar de um estudo multicêntrico, o ELSA-Brasil teve seu protocolo de pesquisa aprovado pelos comitês de ética de cada instituição participante, além do Comitê Nacional de Ética em Pesquisa. Cada onda de coleta de dados recebeu aprovação pelo comitê de ética, e todos os participantes assinaram os formulários de consentimento livre e esclarecido. O presente estudo também foi aprovado pelo comitê de ética em pesquisa (CAAE: 33461220.0.0000.5240).

### Desfecho

O controle glicêmico foi avaliado por meio da dosagem da A1C, categorizada como adequada (A1C< 7) ou inadequada (A1C≥ 7).^18^

### Exposição

Os comportamentos de autocuidado avaliados incluíram – consumo de ultraprocessados, consumo de legumes, verduras e frutas, prática de atividade física, adesão ao tratamento farmacológico e tabagismo. As informações sobre o consumo alimentar foram obtidas por meio do questionário semiquantitativo de frequência alimentar (QFA), composto por 114 itens.^21^ O consumo de ultraprocessados foi estimado como o percentual da contribuição calórica desses alimentos na dieta, utilizando a classificação NOVA, e categorizado em quartis.^22,23^

O consumo de legumes, verduras e frutas calculado com base na ingestão diária (em gramas/dia) e categorizado como adequado (≥ 400g/dia) ou inadequado (< 400g/dia).^24^Foram excluídos da análise os participantes com consumo alimentar geral inferior a 600 Kcal/dia ou superior a 6 000 kcal/dia.

A atividade física foi mensurada por autorrelato utilizando o *International Physical Activity Questionnary* (IPAQ)^25,26^A atividade física foi calculada em minutos por semana e classificada, conforme o IPAQ, em atividades de intensidade leve, moderada ou vigorosa.

A adesão ao tratamento farmacológico para o controle do DT2 foi avaliada com base no relato do uso de medicamentos nas duas semanas anteriores à coleta de dados. O uso por menos de 11 dias foi considerado baixa adesão (aderência < 80%), enquanto o uso por 12 ou mais dias foi considerado alta adesão (aderência ≥ 80%).^27^

O tabagismo foi avaliado por meio de questionário padrão do ELSA-Brasil, e os participantes foram classificados como “não fumante”, “fumante atual” e “ex-fumante”.^26^

### Covariáveis

As covariáveis foram avaliadas por meio de questionários padronizados do ELSA-Brasil e incluíram: idade, sexo, escolaridade, raça/cor autorreferida, situação conjugal, história familiar de diabetes, consumo de álcool e o tipo de terapia farmacológica para DT2.^13,28-30^ Os parâmetros antropométricos (peso corporal e a estatura) foram mensurados utilizando técnicas e equipamentos padronizados, e o Índice de Massa Corporal (IMC) foi calculado a partir dessas medidas.^17^ O IMC e a idade foram tratados como variáveis contínuas. A escolaridade foi classificada em ensino fundamental completo, ensino médio completo e ensino superior completo ou pós-graduação.

A raça/cor autorreferida foi analisada em três categorias: preta, parda e branca. Os participantes que se autodeclararam como amarelos ou indígenas foram excluídos devido ao baixo percentual (4,7%). A situação conjugal foi classificada em: casado/união estável, solteiro e outros (separado, divorciado e viúvo) e a história familiar de diabetes foi dicotomizada em “presença” ou “ausência”.

O consumo de álcool foi avaliado com base na ingestão semanal. Ingestão inferior a 210g/semana (homens) e 140g/semana (mulheres) foi considerada moderada, enquanto valores iguais ou superiores a esses limites foram considerados excessivos.^26^

O tipo de terapia farmacológica foi classificado em três categorias: nenhum medicamento, medicamentos hipoglicemiantes e terapia combinada (medicamentos hipoglicemiantes e insulina).

### Análises estatísticas

A análise descritiva das variáveis foi conduzida por meio de regressão logística simples, adotando um nível de significância estatística de 10% para a seleção das variáveis a serem incluídas no modelo múltiplo. As variáveis de exposição que apresentaram significância estatística na regressão logística simples foram posteriormente analisadas em modelos de regressão logística múltipla.

Os modelos múltiplos foram ajustados pelas covariáveis que demonstraram significância estatística na análise de regressão logística simples, assegurando a inclusão de fatores potenciais de confusão. Para os modelos finais da regressão logística múltipla, foi adotado um nível de significância estatística de 5%.

A qualidade dos modelos estatísticos foi avaliada utilizando o Critério de Informação de Akaike (AIC). Além disso, a adequação do ajuste do modelo final foi verificada por meio do teste de Hosmer-Lemeshow e da análise gráfica dos resíduos de desvio com envelope simulado, assegurando a validade dos resultados. Todas as análises foram realizadas utilizando a linguagem de programação R, versão 4.3.0.

## Resultados

A amostra deste estudo foi composta por 2.567 participantes diagnosticados com DT2, dos quais 52,3% eram do sexo masculino, com idade média de 59,7 ± 8,4 anos. Em relação ao controle glicêmico, 29,3% dos participantes apresentaram níveis inadequados.

A análise revelou que a contribuição calórica média dos alimentos ultraprocessados na dieta correspondia a 22,1 ± 0,09%. A maioria dos participantes atendia às recomendações da Organização Mundial da Saúde (OMS) para o consumo de legumes, verduras e frutas (≥ 400 g/dia), representando 68,7% da amostra; no entanto, 30,7% desses indivíduos ainda apresentavam controle glicêmico inadequado.

Quanto à prática de atividade física, os dados do IPAQ indicaram que 77,4% realizavam atividades leves, 17,6% moderadas e 5% vigorosas. Entre os participantes com controle glicêmico inadequado, 30,5% praticavam atividades leves, 25,6% moderadas e 21,9% vigorosas.

Em relação à adesão ao tratamento farmacológico, 94,6% da amostra total foi considerada com alta adesão, mas 35,5% destes ainda apresentaram controle glicêmico inadequado. O tabagismo foi relatado por 10,7% dos participantes, enquanto 38% eram ex-fumantes. Entre os participantes com controle glicêmico inadequado, 35,3% eram fumantes e 28,1% ex-fumantes.

A regressão logística simples (Tabela 1) indicou que, entre os comportamentos de autocuidado, apenas o consumo de ultraprocessados, o consumo de legumes, verduras e frutas e a adesão ao tratamento farmacológico foram significativamente associados ao controle glicêmico inadequado. Entre as covariáveis, idade, escolaridade, raça/cor autorreferida, história familiar de diabetes e tipo de terapia farmacológica também apresentaram significância estatística.

**Tabela 1:** – Regressão logística simples da associação entre os comportamentos de autocuidado para o manejo de diabetes tipo 2, covariáveis e o controle glicêmico inadequado. ELSA-Brasil, 2012-2014

Variáveis	Controle glicêmico inadequado OR (IC 90%)
**Consumo de ultraprocessados**	
1 quartil (0 - 15%)^a^	Referência
2 quartil (15 - 21%)^a^	1,11 (0,82-1,49)
3 quartil (21 - 28%)^a^	**1,41 (1,04-1,91)**
4 quartil (28 - 65%)^a^	1,18 (0,88-1,59)
**Consumo de legumes, verduras e frutas**	
≥ 400 g/dia	Referência
< 400 g/dia	**1,31 (1,04-1,66)**
**Atividade física**	
vigorosa	Referência
moderada	1,11 (0,61- 1,95)
leve	0,90 (0,52-1,51)
**Adesão ao tratamento farmacológico**	
alta adesão	Referência
baixa adesão	**2,14 (1,18-4,17)**
**Tabagismo**	
não fumante	Referência
ex-fumante	1,09 (0,87-1,35)
fumante atual	0,87 (0,61-1,23)
Idade*	1,01 (1,00-1,03)
Sexo*	
masculino	Referência
feminino	1,09 (0,88-1,35)
Escolaridade*	
superior ou pós-graduação completos	Referência
médio completo	**0,49 (0,35-0,69)**
fundamental completo	**0,56 (0,47-0,74)**
Raça/cor autorreferida*	
branca	Referência
preta	**0,56 (0,42-0,73)**
parda	**0,61 (0,48-0,80)**
Situação conjugal*	
casado ou vive em união	Referência
solteiro	0,82 (0,60-1,11)
outros	0,97 (0,75-1,26)
História familiar de diabetes*	
não	Referência
sim	**0,71 (0,57-0,88)**
IMC*	1,00 (1,00-1,00)
Consumo de álcool*	
não consome atualmente	Referência
consumo moderado^b^	0,92 (0,67-1,26)
consumo excessivo^c^	1,12 (0,82-1,53)
Tipo de terapia farmacológica*	
medicamentos hipoglicemiantes	Referência
terapia combinada	**0,25 (0,14-0,43)**
nenhum medicamento	**1,38 (1,07-1,78)**

*Covariáveis. ^a^Estimativa de contribuição calórica do consumo de ultraprocessados na dieta. ^b^Consumo moderado: ≤ 210g de álcool puro/semana (homem) e ≤ 140g (mulher). ^c^Consumo excessivo: > 210g de álcool puro/semana (homem) e >140g (mulher). AIC: Akaike Information Criterion.

Nos modelos múltiplos ajustados (Tabela 2), a associação entre o consumo de ultraprocessados e o controle glicêmico inadequado foi significativa apenas no terceiro quartil do modelo ajustado pela idade, com um aumento de 44% na chance de controle glicêmico inadequado em comparação ao primeiro quartil (OR: 1,44; IC 95%: 1,06–1,95). No entanto, essa significância não se manteve nos modelos mais ajustados (modelo 5, AIC = 1874,13).

**Tabela 2:** – Regressão logística múltipla da associação entre o consumo de ultraprocessados, consumo de legumes, verduras e frutas, adesão ao tratamento farmacológico e o controle glicêmico inadequado em participantes do ELSA-Brasil diagnosticados com diabetes tipo 2, 2012-2014

Consumo de ultraprocessados	Controle glicêmico inadequado OR (IC 95%)				
	Modelo 1	Modelo 2	Modelo 3	Modelo 4	Modelo 5
1 quartil	1	1	1	1	1
2 quartil	1,13 (0,84-1,52)	1,08 (0,80-1,46)	1,04 (0,77-1,41)	1,02 (0,75-1,38)	0,99 (0,73-1,35)
3 quartil	**1,44 (1,06-1,95)**	1,33 (0,98-1,82)	1,29 (0,95-1,76)	1,31 (0,96-1,79)	1,27 (0,93-1,74)
4 quartil	1,21 (0,90-1,64)	1,10 (0,81-1,49)	1,02 (0,75-1,39)	1,01 (0,74-1,39)	1,00 (0,73-1,37)
AIC	1943,84	1923,03	1916,10	1878,94	1874,13
**Consumo de legumes, verduras e frutas**					
≥ 400 g/dia	1	1	1	1	1
< 400 g/dia	1,33 (1,05-1,69)	1,30 (1,03-1,65)	1,26 (0,99-1,60)	1,24 (0,97-1,58)	1,22 (0,96-1,56)
AIC	1939,78	1917,97	1911,82	1875,5	1870,78
**Adesão ao tratamento farmacológico**					
alta adesão	1	1	1	1	1
baixa adesão	**2,19 (1,20-4,26)**	**2,16 (1,18-4,22)**	**2,32 (1,27-4,55)**	**2,14 (1,16-4,21)**	**2,09 (1,14-4,10)**
AIC	1932,23	1908,85	1900,58	1870,67	1865,72

Modelo 1: Ajustado pela idade; Modelo 2: Ajustado pela idade e escolaridade; Modelo 3: Ajustado pela idade, escolaridade e raça/cor autorreferida; Modelo 4: Ajustado pela idade, escolaridade, raça/cor autorreferida e tipo de terapia farmacológica; Modelo 5: Ajustado pela idade, escolaridade, raça/cor autorreferida, tipo de terapia farmacológica e história familiar de diabetes. Fonte: Elaborado pelo autor.

Em relação ao consumo de legumes, verduras e frutas, os participantes que consumiam menos de 400 g/dia apresentaram maior chance de controle glicêmico inadequado nos modelos ajustados pela idade (OR: 1,33; IC 95%: 1,05–1,69) e pela idade e escolaridade (OR: 1,30; IC 95%: 1,03–1,65). No entanto, essa associação não se manteve significativa nos modelos finais mais ajustados (modelo 5, AIC = 1870,78).

Por fim, as análises dos modelos ajustados para a adesão ao tratamento farmacológico mostraram associação significativa em todos os modelos, ocorrendo uma redução de apenas 10% entre o modelo 1 e o modelo 5. No modelo final (modelo 5, AIC = 1865,72), participantes com baixa adesão apresentaram 109% mais chances de controle glicêmico inadequado em comparação aos participantes com alta adesão (OR: 2,09; IC 95%: 1,14–4,10).

A Figura Central apresenta o tamanho do efeito de cada modelo final para os diferentes comportamentos de autocuidado em relação ao controle glicêmico inadequado.

## Discussão

Os achados deste estudo destacam a relevância dos comportamentos de autocuidado no controle glicêmico de indivíduos com DT2. Os resultados das análises múltiplas indicaram que apenas a variável adesão ao tratamento farmacológico apresentou associação significativa consistente com o controle glicêmico inadequado em todos os modelos ajustados. Indivíduos com baixa adesão ao tratamento apresentaram mais que o dobro de chances de apresentar controle glicêmico inadequado quando comparados aos que aderiam ao tratamento, corroborando com a literatura que destaca a adesão farmacológica adequada como um dos pilares do manejo eficaz do DT2.^27,31,32^

A baixa adesão à medicação é um problema significativo no tratamento do DT2, com estudos mostrando que apenas 54% dos pacientes aderem aos seus medicamentos antidiabéticos orais.^33^ Essa baixa adesão está associada ao controle glicêmico inadequado, aumento do risco de complicações e maiores taxas de mortalidade.^34^ Um estudo descobriu que 61% dos pacientes tiveram baixa adesão ao tratamento farmacológico, com apenas 30% alcançando controle glicêmico ideal.^35^ Os fatores que contribuem para a baixa adesão incluem a complexidade do tratamento, os efeitos adversos e as percepções dos pacientes sobre a ineficácia do tratamento.^36^ É importante ressaltar que uma boa adesão está associada a um melhor controle glicêmico, com pacientes com alta adesão apresentando 1,33 vezes mais chances de atingir um bom controle em comparação aos pacientes com baixa adesão.^33,37^ Para melhorar os resultados, os profissionais de saúde devem se concentrar na educação do paciente, em terapias personalizadas e na abordagem de barreiras à adesão.^34,36^

As associações entre o consumo de alimentos ultraprocessados e o controle glicêmico inadequado permaneceram significativas apenas no modelo ajustado por idade, sugerindo que outros fatores, como perfil socioeconômico e hábitos de vida, podem amenizar esse efeito em modelos mais ajustados. Apesar disso, os alimentos ultraprocessados são amplamente reconhecidos como prejudiciais ao controle metabólico, dado o seu alto conteúdo calórico e baixa densidade nutricional.^38^ Esses alimentos contribuem para o ganho de peso e piora dos níveis glicêmicos, sendo essencial sua redução na dieta de indivíduos com DT2.^22,39,40^

Pesquisas sugerem que o maior consumo de alimentos ultraprocessados está associado a maiores riscos à saúde.^38^ Um grande estudo prospectivo evidenciou que a maior ingestão de alimentos ultraprocessados estava ligada a um maior risco de DT2.^41^ Em crianças, o consumo precoce de alimentos ultraprocessados previu aumento da obesidade abdominal da pré-escola à idade escolar.^42^ Entre mulheres grávidas com diabetes preexistente, maior ingestão de alimentos ultraprocessados no terceiro trimestre foi associada a controle glicêmico inadequado e maior ganho de peso gestacional.^12^

Curiosamente, fatores socioeconômicos podem influenciar os padrões de consumo de alimentos ultraprocessados. Uma análise transversal de adultos brasileiros revelou que indivíduos com maior escolaridade, renda e classe social ocupacional tiveram maior contribuição calórica de alimentos ultraprocessados em suas dietas.^43^ Essas descobertas destacam as relações complexas entre o consumo de alimentos ultraprocessados, a saúde metabólica e os fatores socioeconômicos, enfatizando a necessidade de estratégias de saúde pública para limitar a ingestão de alimentos ultraprocessados em diversas populações.

De modo semelhante, o consumo insuficiente de vegetais, verduras e frutas foi associado ao controle glicêmico inadequado nos modelos ajustados para idade (modelo 1) e para idade e nível de escolaridade (modelo 2), porém perdeu significância nos modelos finais. Apesar disso, a literatura ressalta os benefícios desses alimentos, ricos em fibras e micronutrientes, na melhoria da sensibilidade à insulina e no controle glicêmico.^14,44,45^ Evidências científicas indicam que a ingestão insuficiente de frutas e vegetais está associada a um controle glicêmico inadequado em indivíduos com diabetes.^46,47^ Um estudo^46^ demonstrou que indivíduos em situação de insegurança alimentar relataram menor consumo desses alimentos, o que foi correlacionado a um pior controle glicêmico.^46^ Além disso, o estudo EPIC-Norfolk^47^ observou que participantes que raramente consumiam frutas e vegetais folhosos apresentavam níveis mais elevados de A1C em comparação àqueles com maior frequência de consumo.

Em contraste, diversos estudos demonstraram que o aumento da ingestão de fibras, especialmente aquelas provenientes de grãos integrais, frutas e vegetais, contribui para a melhora do controle glicêmico e da sensibilidade à insulina.^14,44,45^ Uma meta-análise indica que uma maior ingestão de frutas e vegetais folhosos está associada a um risco significativamente reduzido de desenvolvimento de DT2.^48^ Outra meta-análise de ensaios clínicos randomizados evidenciou que dietas ricas em fibras promoveram uma redução de 0,55% nos níveis de hemoglobina glicada e de 9,97 mg/dL na glicemia de jejum.^14^ Esses achados ressaltam a importância da incorporação de alimentos ricos em fibras e vegetais na alimentação de indivíduos diagnosticados com diabetes, visando um melhor controle glicêmico.

Este estudo apresenta limitações inerentes ao seu delineamento transversal, que não permite estabelecer relações causais entre comportamentos de autocuidado e controle glicêmico inadequado. Além disso, informações obtidas por meio de autorrelato podem estar sujeitas a viés de memória e desejabilidade social. A perda de significância de algumas associações em modelos mais ajustados sugere a presença de fatores de confusão não mensurados, o que pode ter impactado os resultados.

Os pontos fortes do estudo incluem o uso de dados de uma coorte multicêntrica de larga escala de diferentes regiões do Brasil, com diversos contextos culturais e alimentares, o que permite análises robustas e abrangentes. Além disso, o uso de instrumentos validados para coleta de dados e ajustes para diversas covariáveis minimizam potenciais fontes de viés. Esses resultados fornecem suporte importante para o desenvolvimento de políticas públicas e intervenções voltadas ao manejo do DT2, com foco na promoção da adesão adequada ao tratamento e comportamentos de autocuidado.

## Conclusão

Este estudo evidenciou que, entre os comportamentos de autocuidado avaliados, a variável adesão ao tratamento farmacológico foi o fator mais consistentemente associado ao controle glicêmico inadequado em indivíduos do ELSA-Brasil vivendo com DT2. Participantes com baixa adesão ao tratamento farmacológico apresentaram maior probabilidade de controle glicêmico inadequado, ressaltando a importância de estratégias que promovam o uso regular de medicamentos para o manejo eficaz da doença.

Embora o consumo de ultraprocessados e a ingestão insuficiente de legumes, verduras e frutas tenham apresentado associações iniciais com o controle glicêmico inadequado, essas relações perderam significância nos modelos ajustados, sugerindo a influência de fatores adicionais, como o contexto socioeconômico e hábitos de vida.

Esses resultados reforçam a necessidade de intervenções integradas que combinem educação em saúde, suporte ao autocuidado e políticas públicas voltadas à equidade no acesso a tratamentos, contribuindo para o controle glicêmico e a redução das complicações associadas ao DT2.
