# Supplementary material for: Associação entre Comportamentos de Autocuidado e o Controle Glicêmico Inadequado em Participantes do ELSA-Brasil com Diabetes Tipo 2
Source: Arq Bras Cardiol. 2025 Jun 27;122(6):e20240867. [Article in Portuguese] doi: 10.36660/abc.20240867 (PMC12266605; doi:10.36660/abc.20240867)
Supplement: Translation

# Translation: Association between Self-Care Behaviors and Inadequate Glycemic Control in ELSA-Brasil Participants with Type 2 Diabetes

**DOI:** 10.36660/abc.20240867i

**Authors:** Gisele de Souza Tupinambá, Odaleia Barbosa de Aguiar, Bruno Pereira de Moura, Maria Inês Schmidt, Maria Del Carmen Bisi Molina, Rosane Harter Griep, Maria de Jesus Mendes da Fonseca

**Affiliations:** 1 Brazil Fundação Oswaldo Cruz – Escola Nacional de Saúde Pública Sérgio Arouca – Programa de Epidemiologia em Saúde Pública, Rio de Janeiro, RJ – Brazil; 2 Brazil Universidade do Estado do Rio de Janeiro – Instituto de Nutrição – Departamento de Nutrição Aplicada, Rio de Janeiro, RJ – Brazil; 3 Brazil Universidade Federal do Rio Grande do Sul – Programa de Pós-Graduação em Epidemiologia, Porto Alegre, RS – Brazil; 4 Brazil Universidade Federal do Espírito Santo – Programa de Pós-Graduação em Nutrição e Saúde, Vitória, ES – Brazil; 5 Brazil Fundação Oswaldo Cruz – Instituto Oswaldo Cruz – Laboratório de Educação em Ambiente e Saúde, Rio de Janeiro, RJ – Brazil; 6 Brazil Fundação Oswaldo Cruz – Escola Nacional de Saúde Pública Sérgio Arouca – Departamento de Epidemiologia e Métodos Quantitativos em Saúde, Rio de Janeiro, RJ – Brazil

**Keywords:** Type 2 Diabetes Mellitus, Self Care, Glycemic Control, Medication Adherence, Processed Food

## Abstract

**Background:**

Type 2 diabetes is a prevalent chronic condition, often associated with metabolic and cardiovascular complications.

**Objective:**

This study aimed to evaluate the association between self-care behaviors, such as consumption of ultra-processed foods, vegetables, greens and fruits, physical activity, adherence to pharmacological treatment and smoking, and inadequate glycemic control in ELSA-Brasil participants with type 2 diabetes.

**Methods:**

This is a cross-sectional study with 2,567 individuals, in which the associations between the variables of interest were analyzed by multiple logistic regression (p<0.05).

**Results:**

Adherence to pharmacological treatment was the only behavior consistently associated with inadequate glycemic control. Participants with low adherence were more than twice as likely to have inadequate levels of glycated hemoglobin (OR: 2.09; 95%CI 1.14–4.10). Consumption of ultra-processed foods and insufficient intake of leafy vegetables showed initial associations that did not remain significant after adjustments, suggesting the influence of additional factors, such as socioeconomic conditions.

**Conclusion:**

These results reinforce the need for interventions that combine health education, self-care support and public policies aimed at equality of treatment access, contributing to glycemic control and the reduction of complications associated with type 2 diabetes.

## Introduction

Type 2 diabetes (T2D) is a chronic condition with a high global prevalence, associated with various metabolic and cardiovascular complications that impact quality of life and mortality.^1,2^ It is estimated that more than 529 million people worldwide have diabetes, and T2D accounts for approximately 96% of cases.^3^In Brazil, the situation is equally alarming, with a growing prevalence in recent years, especially among middle-aged adults and the elderly, requiring effective strategies for disease management and control.^4^

Self-care is an important strategy in diabetes treatment, that may contribute to adequate glycemic control, reduction of disease complications, and improvement of quality of life.^5^ Glycated hemoglobin (A1C) is a widely used marker for assessing glycemic control in individuals with diabetes, as it reflects average blood glucose levels over the past two to three months.^6^According to the latest guidelines, an A1C below 7% is considered adequate, whereas values equal to or greater than 7% indicate poor glycemic control, increasing the risk of microvascular and macrovascular complications.^7,8^

Proper management of T2D involves not only the use of hypoglycemic medications but also the adoption of a set of self-care behaviors that are essential for glycemic control and complication prevention.^9^According to the literature, the main self-care behaviors include regular physical activity, adherence to pharmacological treatment, balanced food consumption, and abstinence from smoking.^10,11^ Additionally, the consumption of ultra-processed foods, which are widely available in contemporary society, has been associated with poorer metabolic control,^12^ whereas the intake of fiber-rich foods, such as vegetables, greens, and fruits, positively contributes to glycemic control.^13,14^

Studies evaluating these self-care behaviors in large populations are scarce in Brazil.^13,15,16^The Longitudinal Study of Adult Health, also known as ELSA-Brasil,^17^ is a multicenter cohort that provides a unique opportunity to investigate this relationship in a large-scale sample, allowing researchers to explore how different self-care practices influence glycemic control in individuals diagnosed with T2D.

Given the significant impact of self-care behaviors on the management of T2D and the need for a better understanding of the variables influencing glycemic control, this study aims to evaluate the association between self-care behaviors (consumption of ultra-processed foods, intake of vegetables, greens, and fruits, physical activity, adherence to pharmacological treatment, and smoking) and inadequate glycemic control in participants of the ELSA-Brasil study with T2D.

## Methods

### Study design and sample

This is a cross-sectional observational study that used data from the second wave (2012–2014) of ELSA-Brasil, an ongoing multicenter cohort study investigating the incidence and risk factors for chronic diseases in a Brazilian population.^17^In its baseline (2008–2010), data were collected from 15,105 active and retired employees, of both sexes, aged 35 to 74 years, from six public institutions across different states in Brazil –Oswaldo Cruz Foundation (FIOCRUZ - Rio de Janeiro), University of São Paulo (USP), and the Federal Universities of Bahia (UFBA), Espírito Santo (UFES), Minas Gerais (UFMG), and Rio Grande do Sul (UFRGS).

As for the inclusion criteria, participants had to have a diagnosis of T2D, which was made based on the following information – self-reported diabetes, self-reported use of diabetes medication in the two weeks prior to data collection, abnormal laboratory tests (fasting blood glucose ≥ 126 mg/dL, A1C ≥ 6.5%, or 2-hour post-load glucose ≥ 200 mg/dL).^18^ The exclusion criteria included participants who had undergone bariatric surgery. Studies have shown that post-surgical patients tend to increase their consumption of ultra-processed foods on weekends compared to weekdays and reduce their intake of fruits and vegetables.^19,20^From the 14,014 individuals in the second wave of ELSA-Brasil, 2 567 participants diagnosed with T2D were selected for the study sample.

Since this is a multicenter study, the ELSA-Brasil research protocol was approved by the ethics committees of each participating institution, as well as by the National Research Ethics Committee. Each wave of data collection received approval from the ethics committee, and all participants signed informed consent forms. The present study was also approved by the research ethics committee (CAAE: 33461220.0.0000.5240).

### Outcome

Glycemic control was assessed by measuring A1C levels, categorized as adequate (A1C < 7) or inadequate (A1C ≥ 7).^18^

### Exposure

The self-care behaviors evaluated included – consumption of ultra-processed foods, intake of vegetables, greens, and fruits, physical activity, adherence to pharmacological treatment, and smoking. Dietary intake information was collected through a semi-quantitative food frequency questionnaire (FFQ), consisting of 114 items.^21^ The consumption of ultra-processed foods was estimated as the percentage of caloric contribution from these foods in the diet, using the NOVA classification, and categorized into quartiles.^22,23^

The intake of vegetables, greens, and fruits was calculated based on daily consumption (in grams/day) and classified as adequate (≥ 400g/day) or inadequate (< 400g/day).^24^ Participants with total daily caloric intake below 600 Kcal/day or above 6,000 kcal/day were excluded from the analysis.

Physical activity was self-reported using the International Physical Activity Questionnaire (IPAQ).^25,26^ Physical activity was calculated in minutes per week and classified, according to IPAQ, into light, moderate, or vigorous in intensity.

Adherence to pharmacological treatment for T2D control was assessed based on self-reported medication use in the two weeks prior to data collection. Usage for less than 11 days was considered low adherence (< 80%), while usage for 12 or more days was considered high adherence (≥ 80%).^27^

Smoking status was evaluated using the standard ELSA-Brasil questionnaire, and participants were classified as “non-smoker,” “current smoker,” or “former smoker.”^26^

### Covariables

The covariates were assessed using standardized ELSA-Brasil questionnaires and included: age, sex, education level, self-reported race/color, marital status, family history of diabetes, alcohol consumption, and type of pharmacological therapy for T2D.^13,28-30^Anthropometric parameters (body weight and height) were measured using standardized techniques and equipment, and body mass index (BMI) was calculated based on these measurements.^17^ BMI and age were treated as continuous variables. Education level was categorized into completed elementary school, completed high school, and completed higher education / postgraduate degree.

Self-reported race/color was analyzed in three categories: Black, Brown, and White. Participants who self-identified as Yellow or Indigenous were excluded due to the low percentage (4.7%). Marital status was classified as married/stable union, single, and other (separated, divorced, or widowed). Family history of diabetes was dichotomized as “present” or “absent”.

Alcohol consumption was assessed based on weekly intake. Intake below 210g/week for men and 140g/week for women was considered moderate, while values equal to or exceeding these limits were classified as excessive.^26^

Pharmacological therapy was classified into three categories: no medication, hypoglycemic medications, and combination therapy (hypoglycemic medications and insulin).

### Statistical analysis

Descriptive analysis of the variables was conducted using simple logistic regression, adopting a statistical significance level of 10% for selecting variables to be included in the multiple model. Exposure variables that showed statistical significance in the simple logistic regression were subsequently analyzed using multiple logistic regression models.

The multiple models were adjusted for covariates that demonstrated statistical significance in the simple logistic regression analysis, ensuring the inclusion of potential confounding factors. For the final multiple logistic regression models, a statistical significance level of 5% was adopted.

The quality of the statistical models was assessed using the Akaike Information Criterion (AIC). Additionally, the adequacy of the final model fit was verified through the Hosmer-Lemeshow test and graphical analysis of deviance residuals with a simulated envelope, ensuring the validity of the results.

All analyses were performed using the R programming language, version 4.3.0.

## Results

The sample for this study consisted of 2,567 participants diagnosed with T2D, of whom 52.3% were male, with a mean age of 59.7 ± 8.4 years. Regarding glycemic control, 29.3% of participants had inadequate blood glucose levels.

The analysis revealed that the average caloric contribution from ultra-processed foods in the diet was 22.1 ± 0.09%. Most participants met the World Health Organization (WHO) recommendations for vegetable, greens, and fruit consumption (≥ 400 g/day), accounting for 68.7% of the sample; however, 30.7% of these individuals still had inadequate glycemic control.

Regarding physical activity, IPAQ data indicated that 77.4% engaged in light activities, 17.6% in moderate activities, and 5% in vigorous activities. Among participants with inadequate glycemic control, 30.5% practiced light activities, 25.6% moderate, and 21.9% vigorous.

Regarding adherence to pharmacological treatment, 94.6% of the total sample was considered to have high adherence, yet 35.5% of these individuals still exhibited inadequate glycemic control. Smoking was reported by 10.7% of participants, while 38% were former smokers. Among those with inadequate glycemic control, 35.3% were smokers and 28.1% former smokers.

Simple logistic regression analysis (Table 1) indicated that among self-care behaviors, only consumption of ultra-processed foods, intake of vegetables, greens, and fruits, and adherence to pharmacological treatment were significantly associated with inadequate glycemic control. Among the covariates, age, education level, self-reported race/color, family history of diabetes, and type of pharmacological therapy also showed statistical significance.

**Table 1:** – Simple logistic regression of the association between self-care behaviors for Type 2 Diabetes (T2D) management, covariates, and inadequate glycemic control; ELSA-Brasil, 2012-2014

Variables	Adequate glycemic control OR (90%CI)
**Ultra-processed food consumption**	
1st quartile (0 - 15%)^a^	Reference
2nd quartile (15 - 21%)^a^	1.11 (0.82-1.49)
3rd quartile (21 - 28%)^a^	**1.41 (1.04-1.91)**
4th quartile (28 - 65%)^a^	1.18 (0.88-1.59)
**Vegetables, greens and fruit consumptions**	
≥ 400 g/day	Reference
< 400 g/day	**1.31 (1.04-1.66)**
**Physical activity**	
Vigorous	Reference
Moderate	1.11 (0.61- 1.95)
Mild	0.90 (0.52-1.51)
**Adherence to pharmacological treatment**	
High adherence	Reference
Low adherence	**2.14 (1.18-4.17)**
**Smoking**	
Non-smoker	Reference
Former smoker	1.09 (0.87-1.35)
Current smoker	0.87 (0.61-1.23)
Age*	1.01 (1.00-1.03)
Sex*	
Male	Reference
Female	1.09 (0.88-1.35)
Education level *	
Higher education/postgraduate degree	Reference
High school	**0.49 (0.35-0.69)**
Elementary school	**0.56 (0.47-0.74)**
Self-reported race/skin color*	
White	Reference
Black	**0.56 (0.42-0.73)**
Pardo	**0.61 (0.48-0.80)**
Marital status*	
Married/stable union	Reference
Single	0.82 (0.60-1.11)
others	0.97 (0.75-1.26)
Family history of diabetes *	
No	Reference
Yes	**0.71 (0.57-0.88)**
BMI*	1.00 (1.00-1.00)
Alcohol intake*	
No intake	Reference
Moderate intake^b^	0.92 (0.67-1.26)
Excess intake^c^	1.12 (0.82-1.53)
Pharmacological therapy*	
Hypoglycemic medications	Reference
Combined therapy	**0.25 (0.14-0.43)**
No medication	**1.38 (1.07-1.78)**

*Covariables. ^a^Caloric contribution from ultra-processed foods to the diet. ^b^Moderate intake: ≤ 210g pure alcohol/week (men) and ≤ 140g (women). ^c^Excess intake: > 210g pure alcohol/week (men) and > 140g (women).

In the adjusted multiple models (Table 2), the association between ultra-processed food consumption and inadequate glycemic control was significant only in the third quartile of the age-adjusted model, with a 44% increase in the likelihood of inadequate glycemic control compared to the first quartile (OR: 1.44; 95% CI: 1.06–1.95). However, this significance did not persist in the more adjusted models (model 5, AIC = 1874.13).

**Table 2:** – Multiple logistic regression of the association between ultra-processed food consumption, intake of vegetables, greens, and fruits, adherence to pharmacological treatment, and inadequate glycemic control in ELSA-Brasil participants diagnosed with type 2 diabetes, 2012-2014

Ultra-processed food consumption	Inadequate glycemic control OR (95%CI)				
	Model 1	Model 2	Model 3	Model 4	Model 5
1^st^ quartile	1	1	1	1	1
2^nd^ quartile	1.13 (0.84-1.52)	1.08 (0.80-1.46)	1.04 (0.77-1.41)	1.02 (0.75-1.38)	0.99 (0.73-1.35)
3^rd^ quartile	**1.44 (1.06-1.95)**	1.33 (0.98-1.82)	1.29 (0.95-1.76)	1.31 (0.96-1.79)	1.27 (0.93-1.74)
4^th^ quartile	1.21 (0.90-1.64)	1.10 (0.81-1.49)	1.02 (0.75-1.39)	1.01 (0.74-1.39)	1.00 (0.73-1.37)
AIC	1943.84	1923.03	1916.10	1878.94	1874.13
**Vegetables, greens and fruit intake**					
≥ 400 g/day	1	1	1	1	1
< 400 g/day	1.33 (1.05-1.69)	1.30 (1.03-1.65)	1.26 (0.99-1.60)	1.24 (0.97-1.58)	1.22 (0.96-1.56)
AIC	1939.78	1917.97	1911.82	1875.5	1870.78
**Pharmacological treatment adherence**					
High adherence	1	1	1	1	1
Low adherence	**2.19 (1.20-4.26)**	**2.16 (1.18-4.22)**	**2.32 (1.27-4.55)**	**2.14 (1.16-4.21)**	**2.09 (1.14-4.10)**
AIC	1932.23	1908.85	1900.58	1870.67	1865.72

Model 1: Adjusted for age; Model 2: Adjusted for age and education level; Model 3: Adjusted for education level and self-reported race/skin color; Model 4: Adjusted for age, education level, self-reported race/skin color and type of pharmacological therapy; AIC: Akaike Information Criterion. Model 5: Adjusted for age, education level, self-reported race/skin color and type of pharmacological therapy and family history of diabetes. Source: the authors.

Regarding vegetable, greens, and fruit intake, participants consuming less than 400 g/day had a higher likelihood of inadequate glycemic control in age-adjusted models (OR: 1.33; 95% CI: 1.05–1.69) and in models adjusted for age and education level (OR: 1.30; 95% CI: 1.03–1.65). However, this association did not remain significant in the final, more adjusted models (model 5, AIC = 1870.78).

Finally, analyses of the adjusted models for adherence to pharmacological treatment showed a significant association across all models, with only a 10% reduction between model 1 and model 5. In the final model (model 5, AIC = 1865.72), participants with low adherence had a 109% higher chance of inadequate glycemic control compared to those with high adherence (OR: 2.09; 95% CI: 1.14–4.10).

The Central Figure illustrates the effect size of each final model for the different self-care behaviors in relation to inadequate glycemic control.

## Discusssion

The findings of this study highlight the importance of self-care behaviors in glycemic control among individuals with T2D. The results of multiple analyses indicated that only adherence to pharmacological treatment showed a consistent significant association with inadequate glycemic control across all adjusted models. Individuals with low adherence to treatment had more than twice the odds of experiencing inadequate glycemic control compared to those who adhered to treatment, supporting the literature emphasizing adequate pharmacological adherence as a key pillar of effective T2D management.^27,31,32^

Low medication adherence is a significant issue in T2D treatment, with studies showing that only 54% of patients adhere to their oral antidiabetic medications.^33^ This low adherence is associated with inadequate glycemic control, increased risk of complications, and higher mortality rates.^34^One study found that 61% of patients had low adherence to pharmacological treatment, with only 30% achieving optimal glycemic control.^35^ Factors contributing to low adherence include treatment complexity, adverse effects, and patient perceptions of treatment inefficacy.^36^It is important to emphasize that good adherence is associated with better glycemic control; highly adherent patients had a 1.33 greater likelihood of achieving good glycemic control compared to those with low adherence.^33,37^To improve outcomes, healthcare professionals should focus on patient education, personalized therapies, and addressing barriers to adherence.^34,36^

The associations between ultra-processed food consumption and inadequate glycemic control remained significant only in the age-adjusted model, suggesting that other factors, such as socioeconomic profile and lifestyle habits, may mitigate this effect in more adjusted models. Despite this, ultra-processed foods are widely recognized as harmful to metabolic control due to their high caloric content and low nutritional density.^38^ These foods contribute to weight gain and worsening glycemic levels, making their reduction essential in the diet of T2D individuals.^22,39,40^

Research suggests that higher consumption of ultra-processed foods is associated with greater health risks.^38^ A large prospective study showed that higher intake of ultra-processed foods was linked to a higher risk of T2D.^41^In children, early consumption of ultra-processed foods was found to predict an increase in abdominal obesity from preschool to school age.^42^ Among pregnant women with preexisting diabetes, higher intake of ultra-processed foods in the third trimester was associated with inadequate glycemic control and greater gestational weight gain.^12^

Interestingly, socioeconomic factors may influence the patterns of ultra-processed food consumption. A cross-sectional analysis of Brazilian adults revealed that individuals with higher education, income, and occupational social class had higher caloric contributions from ultra-processed foods in their diets.^43^These findings highlight the complex relationships between ultra-processed food consumption, metabolic health, and socioeconomic factors, reinforcing the need for public health strategies to limit the intake of ultra-processed foods across different populations.

Similarly, insufficient consumption of vegetables, greens, and fruits was associated with inadequate glycemic control in models adjusted for age (Model 1) and age and education level (Model 2), but lost significance in the final models. Despite this, the literature highlights the benefits of these fiber-rich and micronutrient-dense foods in improving insulin sensitivity and glycemic control.^14,44,45^Scientific evidence indicates that insufficient intake of fruits and vegetables is associated with inadequate glycemic control in individuals with diabetes.^46,47^One study^46^ showed that individuals experiencing food insecurity reported lower consumption of these foods, which was correlated with poorer glycemic control.^46^ Additionally, the EPIC-Norfolk study^47^ observed that participants who rarely consumed fruits and leafy vegetables had higher A1C levels compared to those who consumed them more frequently.

In contrast, several studies have shown that increased fiber intake, especially from whole grains, fruits, and vegetables, contributes to improved glycemic control and insulin sensitivity.^14,44,45^A meta-analysis^48^ indicates that higher consumption of fruits and leafy vegetables is associated with a significantly reduced risk of developing T2D. Another meta-analysis^14^ of randomized clinical trials demonstrated that fiber-rich diets led to a 0.55% reduction in glycated hemoglobin levels and a 9.97 mg/dL decrease in fasting blood glucose. These findings reinforce the importance of incorporating fiber-rich foods and vegetables into the diet of individuals diagnosed with diabetes, aiming for better glycemic control.

This study presents limitations inherent to its cross-sectional design, which does not allow for causal relationships between self-care behaviors and inadequate glycemic control. In addition, information obtained through self-reporting may be subject to memory bias and social desirability bias. The loss of significance of some associations in more adjusted models suggests the presence of unmeasured confounding factors, which may have influenced the results.

The strengths of this study include the use of data from a large-scale multicenter cohort covering different regions of Brazil, with varied cultural and dietary contexts, allowing for robust and comprehensive analyses. Also, the use of validated instruments for data collection and adjustments for multiple covariates minimizes potential sources of bias. These findings provide important support for the development of public policies and interventions aimed at managing T2D, focusing on promoting adherence to treatment and self-care behaviors.

## Conclusion

This study showed that, among the evaluated self-care behaviors, adherence to pharmacological treatment was the factor most consistently associated with inadequate glycemic control in individuals from ELSA-Brasil living with T2D. Participants with low adherence to pharmacological treatment had a higher probability of inadequate glycemic control, emphasizing the importance of strategies that promote regular medication use for the effective management of the disease.

Although ultra-processed food consumption and insufficient intake of vegetables, greens, and fruits initially showed associations with inadequate glycemic control, these relationships lost significance in the adjusted models, suggesting the influence of additional factors, such as socioeconomic context and lifestyle habits.

These results reinforce the need for integrated interventions that combine health education, self-care support, and public policies aimed at equity in access to treatments, contributing to glycemic control and the reduction of complications associated with T2D.
